# Rising burden of early-onset lung cancer among Chinese adolescents and young adults (1990–2023): a Bayesian age-period-cohort analysis with projections to 2038

**DOI:** 10.3389/fmed.2026.1911046

**Published:** 2026-08-27

**Authors:** Yi Dong, Yongjie Guan, Maosen Zhao, Shuang Feng, Xiaowei Wang, Mingming Wang, Maoyong Fu, Jiayu Shang, Ke Gao

**Affiliations:** 1 Department of Cardio-Thoracic Surgery, North Sichuan Medical College, School of Clinical Medicine, Nanchong, Sichuan, China; 2 West China School of Medicine, Sichuan University, Sichuan University affiliated Chengdu Second People's Hospital, Chengdu Second People's Hospital, Chengdu, Sichuan, China; 3 Department of Emergency, West China School of Medicine, Sichuan University, Sichuan University affiliated Chengdu Second People's Hospital, Chengdu Second People's Hospital, Chengdu, Sichuan, China; 4 Department of Cardio-Thoracic Surgery, West China School of Medicine, Sichuan University, Sichuan University affiliated Chengdu Second People's Hospital, Chengdu Second People's Hospital, Chengdu, Sichuan, China; 5 Department of Neurology, West China Hospital of Sichuan University, Chengdu, Sichuan, China

**Keywords:** age–period–cohort analysis, ARIMA projection, BAPC model, China, disease burden, GBD, lung cancer, young adults

## Abstract

**Background:**

Early-onset lung cancer (diagnosed at age 15–39 years) remains uncommon, yet incidence is rising in several world regions. China—the largest contributor to global lung cancer mortality—has not had its youth burden comprehensively characterized or projected.

**Methods:**

Using the Global Burden of Disease Study 2023 (GBD 2023), we extracted annual incidence, prevalence, mortality, and disability-adjusted life-years (DALYs) for Chinese adolescents and young adults (AYA) aged 15–39 years from 1990 to 2023. Joinpoint regression quantified temporal slopes; age–period–cohort (APC) and Bayesian APC (BAPC) models dissected historical drivers. Two complementary forecasting frameworks, BAPC (structural) and autoregressive integrated moving average (ARIMA; time-series) were used to generate burden trajectories to 2038.

**Results:**

Between 1990 and approximately 2020, the age-standardized disease burden generally declined, followed by a recent increase through 2023. Males consistently exhibit a higher disease burden than females; the 35–39-year age band showed the heaviest burden. Age-period-cohort analysis revealed divergent cohort effects: Cohort rate ratios generally declined across successive birth cohorts, with lower risks observed among cohorts born after 1985. For both sexes combined, BAPC point projections indicated that ASIR would increase from 1.53 to 2.54 per 100,000 and the age-standardized DALY rate from 64.71 to 79.77 per 100,000 between 2023 and 2038. These endpoint predictions corresponded to *post hoc* annualized geometric changes of **+**3.44% (UI − 9.37 to +7.83%) and +1.41% (UI − 7.86 to +5.31%), respectively. These estimates describe changes in age-standardized rates rather than absolute incident cases or DALY counts and are not BAPC drift coefficients. In contrast, ARIMA suggested modest declines or relatively flat trajectories for ASIR and ASMR, with wide prediction intervals. The discrepancy reflects model philosophy—BAPC extrapolates age-period-cohort structures, whereas ARIMA extrapolates short-term autoregressive patterns.

**Conclusion:**

After decades of decline, lung cancer burden in Chinese adolescents and young adults is creeping upward and may accelerate. Rising computed tomography screening intensity, persistent ambient pollution, evolving tobacco-product use, and hereditary susceptibility warrant urgent scrutiny. These findings inform precision prevention strategies—targeted tobacco control, cleaner-air policies, and risk-stratified early detection—to avert a foreseeable surge in early-onset lung cancer in China.

## 1 Introduction

Lung cancer remains the world’s deadliest malignancy. For decades, it has been framed as a disease of older age: risk climbs sharply after 50 years, and the median age at diagnosis in the United States is 71 years (1). Yet beneath this familiar narrative, a quieter epidemic is emerging. In every global region, including those with aggressive tobacco-control policies, clinics are reporting more patients whose first symptoms appear before their fortieth birthday (2).

Although these “early-onset” cases still account for fewer than 2% of all lung cancers, their absolute numbers are rising. In high-income countries, the increase is concentrated among women and never-smokers; in low- and middle-income countries, the pattern is driven by ambient air pollution, household solid-fuel use, and changing smoking behaviors. East Asia now carries the heaviest burden of lung cancer in 15- to 49-year-olds, and China sits at the epicenter (3). But granular, age-specific trends for adolescents and young adults (AYA; 15–39 years) have not been published. In this study, we focused on adolescents and young adults (AYA) aged 15–39 years. Although “early-onset lung cancer” is often defined using broader age cutoffs such as <50 or <55 years, the present analysis uses the AYA definition to specifically characterize lung cancer burden in individuals aged 15–39 years.

This evidence gaps matters. AYA patients face unique biology—more adenocarcinoma, advanced stage at presentation, longer symptom duration—and unique psychosocial costs: interrupted education, lost labor, and dependent children. Whether China’s AYA lung cancer burden is growing, stabilizing, or declining is therefore more than an academic question; it determines how aggressively policymakers should target tobacco, pollution, and early-detection programs at the country’s 400 million young adults.

Using the newly released GBD 2023, we quantify incidence, mortality, and DALYs attributable to lung cancer in Chinese aged 15–39 years from 1990 to 2023. We decompose drivers with age–period–cohort models, then project plausible trajectories to 2038 under two complementary forecasting frameworks. The findings provide the first national baseline for AYA lung cancer burden in China and inform precision-prevention strategies aimed at averting a new wave of avoidable premature mortality.

## 2 Methods

### 2.1 Data source and case definition

All estimates for AYA (15–39 years) were drawn from GBD 2023, the most comprehensive epidemiological database currently available. GBD 2023 provides annual, age-specific counts and rates for 375 diseases and injuries across 204 countries and territories from 1990 to 2023 (4). The resource is publicly accessible through the GBD Results Tool (https://vizhub.healthdata.org/gbd-results/) and GBD 2023 Data Resources (https://ghdx.healthdata.org/gbd-2023); methodological details have been published elsewhere (5).

Within the GBD cause hierarchy, lung cancer sits at Level 3 under the cascade: Non-communicable diseases → Neoplasms → Tracheal, bronchus, and lung cancer. Corresponding International Classification of Diseases, 10th Revision (ICD-10) codes are C33–C34 (primary malignancies of the trachea, bronchus, and lung). Sub-sites are mapped to C34.0–C34.9; secondary thoracic deposits (C78.x) are redistributed to the primary lung category by the GBD garbage-code algorithm.

Data were accessed on October 17, 2025. The extraction parameters were: cause: tracheal, bronchus, and lung cancer (cause_id = 426); locations: China (location_id = 6); years, 1990–2023; sexes: male, female, and both sexes (sex_id = 1, 2, and 3); ages: 15–19, 20–24, 25–29, 30–34, and 35–39 years (age_id = 8–12); measures: deaths, DALYs, prevalence, and incidence (measure_id = 1, 2, 5, and 6); and metrics, number and rate (metric_id = 1 and 3). All underlying input data, modeling steps, and uncertainty distributions are documented in the open GBD 2023 capstone publications and can be downloaded from the Global Health Data Exchange. Because the study uses only de-identified, publicly available estimates, institutional review board approval and individual consent were not required.

### 2.2 Statistical analysis

Descriptive statistics were first generated for absolute counts and crude rates, stratified by sex, 5-year age band (15–19, 20–24, 25–29, 30–34, 35–39 years), and GBD super-region when relevant. Age-standardized rates per 100,000 population (ASR) and their 95% UIs were computed with the GBD 2023 world-standard population (6).

Temporal trends were quantified with Joinpoint regression (version 5.3.0.0; National Cancer Institute, Bethesda, MD, USA) to identify inflection years and to estimate the average annual percent change (AAPC) in incidence and DALYs for the entire 1990–2023 interval.

Forecasts to 2038 were produced with two complementary models:

1. Bayesian age–period–cohort (BAPC) model: All unknown parameters are treated as random variables endowed with weakly informative priors; second-order random-walk priors ensure smoothness. Posterior marginals are obtained by integrated nested Laplace approximation (INLA), eliminating Markov-chain Monte Carlo convergence issues (7, 8). The model was trained on GBD 1990–2023 incidence, mortality, and DALY estimates and used to forecast ASRs through 2038. 2. ARIMA model: After Box–Jenkins identification, the best-fitting autoregressive integrated moving-average model was selected on the basis of minimized Akaike information criterion (AIC) and Ljung–Box residuals (*p* > 0.05, 9].

BAPC model calibration was assessed using conditional predictive ordinate and probability integral transform diagnostics generated by INLA. Temporal predictive performance of the ARIMA models was evaluated using expanding-window rolling-origin validation. The initial training period was 1990–2017; for each target year from 2018 to 2023, the model was refitted using all observations available through the preceding year and used to generate a one-step-ahead forecast. Predictive accuracy was summarized using the mean absolute error, root mean squared error, and empirical coverage of the 95% prediction intervals. All analyses were conducted in R version 4.5.0 (R Foundation for Statistical Computing, Vienna, Austria) using the BAPC, INLA, and forecast packages (BAPC 0.0.36, INLA 24.12.11, and forecast 9.0–2). Two-sided *p*-values < 0.05 were considered statistically significant.

#### 2.2.1 Age standardization

Age-standardized rates (ASR) for the 15–39-year population were computed with the direct method (9):

ASR = *Σ* (rᵢ × wᵢ) / Σ wᵢ.

where rᵢ = incidence (or mortality/DALY) rate in the ith 5-year age stratum (15–19, 20–24, 25–29, 30–34, 35–39 years), and wᵢ = weight of the ith stratum in the GBD 2023 world-standard population. The weights for these five age groups were restricted to the 15–39-year range and renormalized to sum to 1. The resulting normalized weights were 0.2191, 0.2062, 0.2007, 0.1935, and 0.1805, respectively. The resulting ASRs were expressed per 100,000 population.

#### 2.2.2 Joinpoint regression

Joinpoint regression was used to track how lung cancer burden among Chinese AYA evolved from 1990 to 2023. This piece-wise linear model, fitted to the log-transformed age-standardized rates, identifies years in which the slope of the trend changes and estimates both the annual percent change (APC) for each segment and the overall AAPC for the entire period (10). The optimal number and location of inflection points were selected by permutation tests with Bonferroni adjustment, preserving an overall significance level of 0.05. Least-squares estimation minimized the residual sum of squares, removing the subjectivity inherent in single-line trend analyses. Statistical significance of the AAPC was judged by a two-sided test against zero, with *p* < 0.05 considered significant. Joinpoint analyses were conducted using the Joinpoint Regression Program, version 5.3.0.0 (National Cancer Institute, USA).

#### 2.2.3 Age-period-cohort analysis

Age–period–cohort (APC) analysis was performed in R version 4.5.0 using the data.table package (version 1.16.4) for data processing, ggplot2 (version 3.5.1) and cowplot (version 1.1.3) for visualization, and the local R implementation of the NCI APC Web Tool described by Rosenberg et al. (11). The analysis included Chinese individuals of both sexes aged 15–39 years and was conducted separately for lung cancer incidence and DALY rates.

The five-year age groups were 15–19, 20–24, 25–29, 30–34, and 35–39 years. Calendar years were grouped into the following diagnostic periods: 1990–1993, 1994–1998, 1999–2003, 2004–2008, 2009–2013, 2014–2018, and 2019–2023. Within each age–period cell, the GBD-estimated event counts and population denominators were averaged across the corresponding years. The age-specific rate was then calculated as the averaged event count divided by the averaged population and expressed per 100,000 population.

The grouped event and population matrices were prepared using the rates() function and fitted using the apc2fit() function. The APC implementation uses a Holford-type parameterization to address the intrinsic linear dependency among age, period, and cohort (11, 12). Accordingly, inference focused on identifiable estimable functions, including longitudinal and cross-sectional age curves, period rate ratios, cohort rate ratios, fitted temporal trends, local drifts, and age-, period-, and cohort-specific deviations. The separate raw linear components of age, period, and cohort were not interpreted independently. Estimates were reported with model-based 95% confidence intervals.

#### 2.2.4 Bayesian age–period–cohort (BAPC) model

To project the future lung cancer burden among Chinese individuals aged 15–39 years, we fitted Bayesian age–period–cohort (BAPC) models using the BAPC package in R, following established Bayesian APC projection methods (7, 8). Models were estimated using integrated nested Laplace approximation (INLA) within a constrained latent Gaussian framework (13). Age, period, and cohort effects were specified as intrinsic second-order random walks (RW2) with penalized smoothness priors and the default sum-to-zero identifiability constraints implemented by BAPC/INLA (constr = TRUE). Because the separate linear components of age, period, and cohort remain non-identifiable, they were not interpreted; inference focused on identifiable fitted and projected age-specific and age-standardized rates (12).

All BAPC outputs were reported as rates per 100,000 population rather than absolute numbers of cases, prevalent cases, deaths, or DALYs. Age-standardized rates used the renormalized GBD 2023 standard-population weights for the 15–39-year age range. Analyses were conducted using R 4.5.0, BAPC 0.0.36, and INLA 24.12.11. Default BAPC priors were applied, including log-Gamma (1, 5 × 10^−5^) precision priors for the RW2 effects and log-Gamma (1, 0.005) for the iid overdispersion term. Fifteen-year projections were generated using npredict = 15 and retro = FALSE. Model fit was assessed using DIC, effective number of parameters, marginal log-likelihood, and CPO diagnostics. Because INLA uses deterministic approximation rather than MCMC, conventional MCMC convergence diagnostics were not applicable (13). Sensitivity analyses refitted the models using scaled RW2 priors, removal of the iid overdispersion term, or removal of the cohort effect, while retaining the same identifiability constraints. Projected 2038 age-standardized rates and their direction of change from 2023 to 2038 were compared with the main model; directional consistency was defined as agreement in the direction of change. The 95% UIs were derived from INLA-approximated posterior marginal distributions and interpreted as Bayesian credible intervals (7). Annualized geometric changes were calculated *post hoc* from the 2023-to-2038 rate ratios; their uncertainty intervals were derived by applying the same transformation to the lower and upper bounds of the corresponding 2038 95% UI.

#### 2.2.5 ARIMA model

To generate complementary projections for the period 2023–2038, standard ARIMA(p, d, q) models were fitted to age-standardized rate (ASR) series, namely incidence and mortality. Model parameters— p (autoregressive order), d (differencing order), and q (moving-average order) were selected using the auto.arima() function from the R forecast package (9.0–2). Candidate models were compared using the Akaike information criterion corrected for small-sample bias (AICc), with stepwise = FALSE and approximation = FALSE to ensure a comprehensive exhaustive search over the candidate parameter space. Model selection was performed separately for each sex and outcome measure, and the specification with the lowest AICc value was adopted for forecasting. The adequacy of the selected models was further assessed using the Ljung–Box test for residual autocorrelation. All models were estimated on the training period 1990–2023 (14).

## 3 Results

### 3.1 Descriptive findings

**Figure 1:**
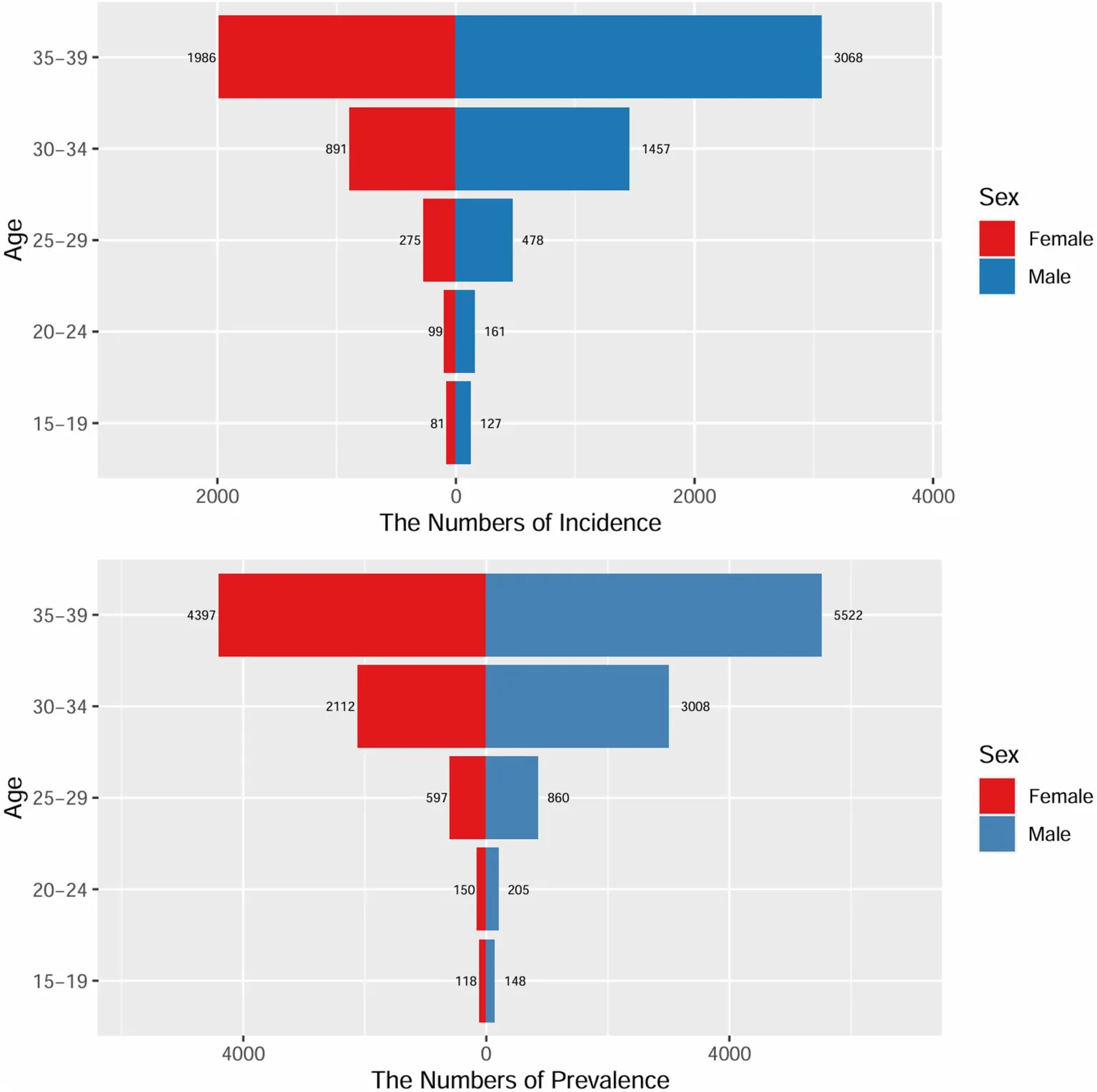
Statistical chart of lung cancer incidence and prevalence cases among males and females aged 15–39 years in China, 2023.

In 2023, every step up the age ladder brought a steep rise in lung cancer caseloads among Chinese AYA. Incident cases climbed from 127 males and 81 females aged 15–19 years to 3,068 males and 1,986 females at 35–39 years; prevalence followed the same gradient, reaching 5,522 males and 4,397 females in the oldest quinquennium (Figure 1). Males outnumbered females at every age level, and the gap widened after age 30, underscoring a clear male and older-age predominance that emerged sharply in 2021 and persisted through 2023.

**Figure 2:**
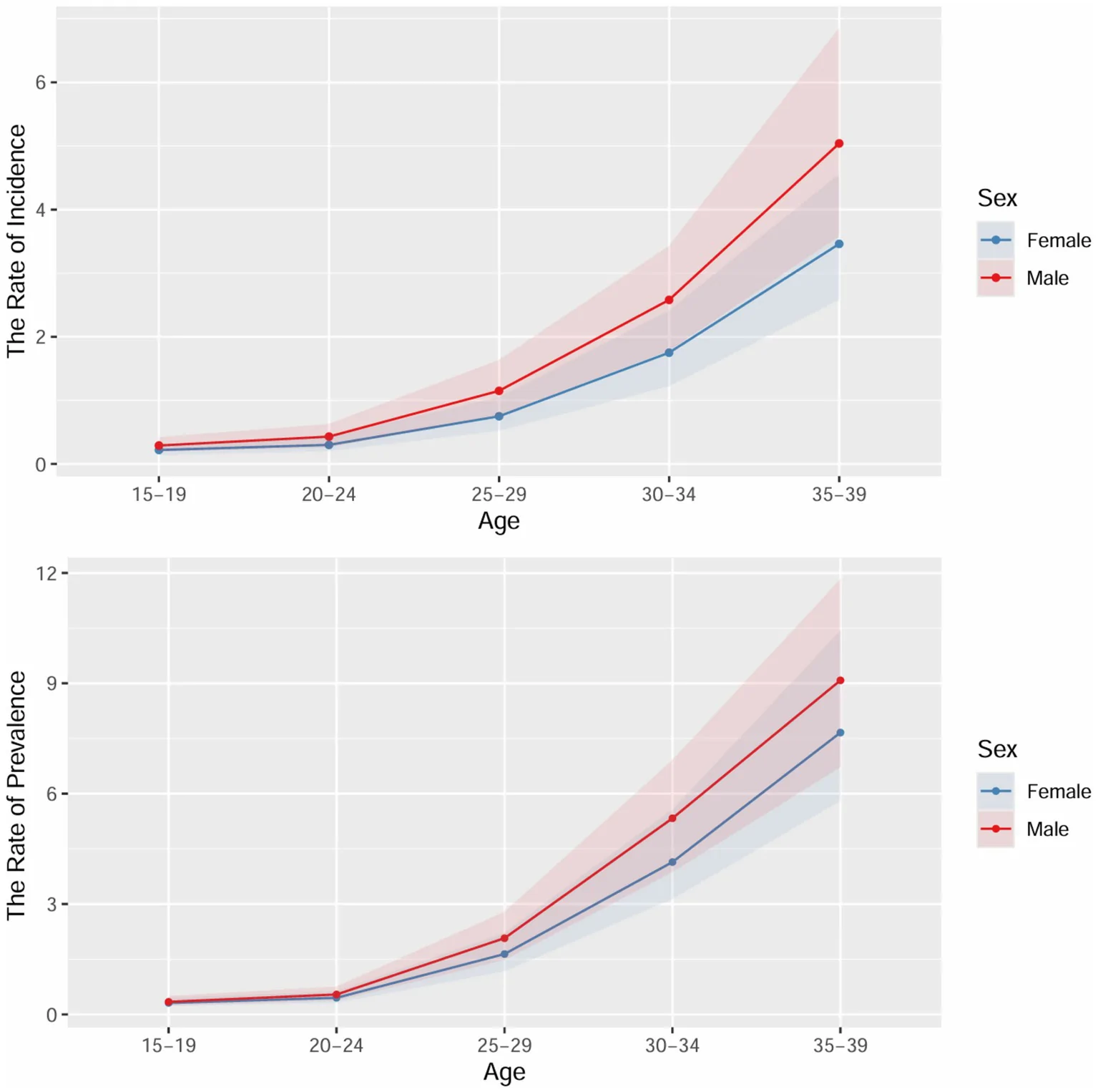
Statistical chart of lung cancer incidence and prevalence rates among males and females aged 15–39 years in China, 2023 (unit: per 100,000 population).

Figure 2 depicts the 2023 sex-specific incidence and prevalence of lung cancer across 5-year age strata among Chinese aged 15–39 years. The curves reveal a persistent male excess and a clear age gradient: rates rise steadily with age, then accelerate after 30 years, with men in the 35–39 bracket showing the steepest upswing. Thus, both incidence and prevalence remained consistently higher in males than in females and in older versus younger AYAs, underscoring a dual burden that intensifies beyond the third decade.

**Figure 3:**
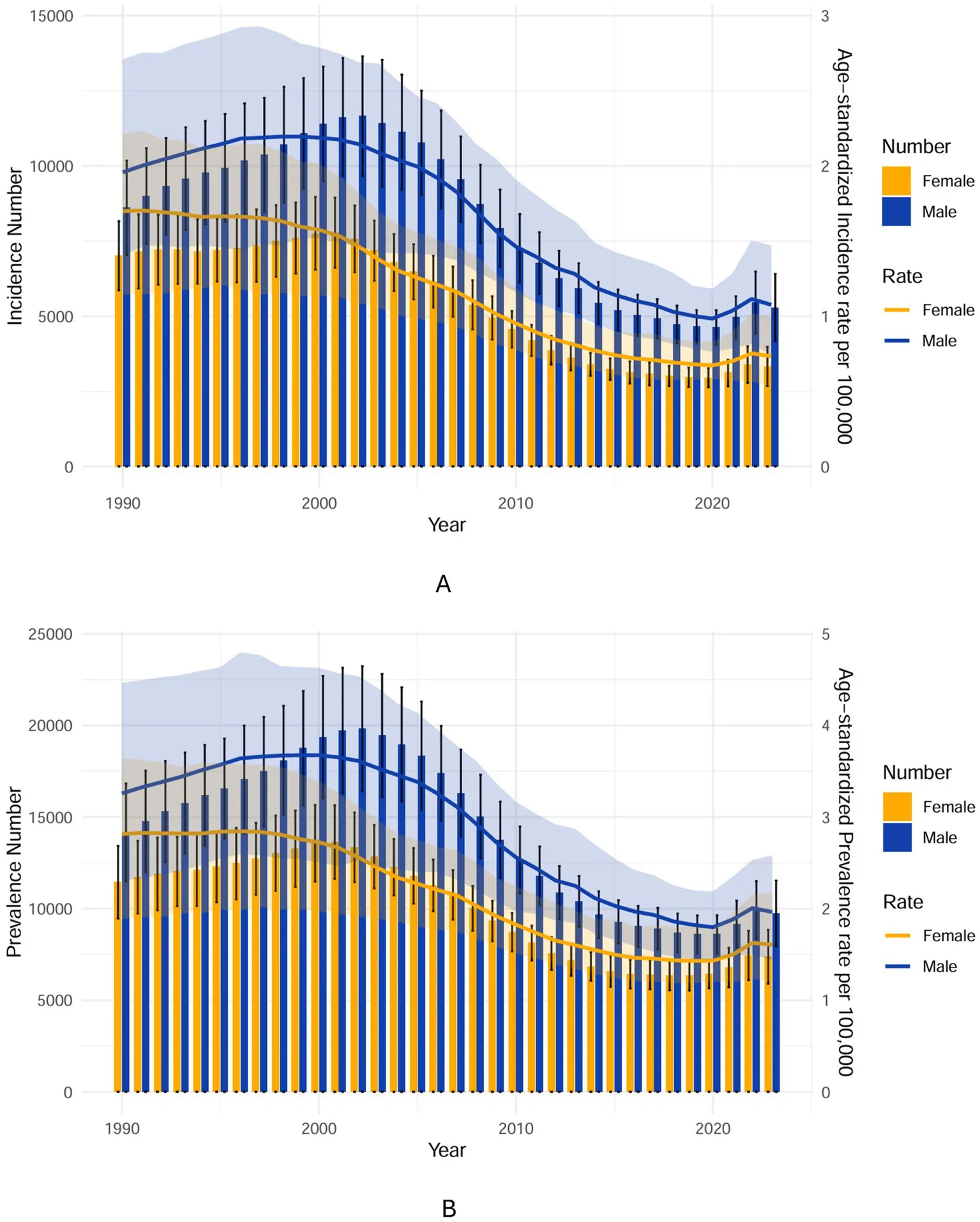
Age-standardized incidence rates/cases **(A)** and prevalence rates/cases **(B)** among the population aged 15–39 years during the period 1990–2023.

Figure 3 traces the sex-specific, age-standardized incidence and prevalence of lung cancer in Chinese 15- to 39-year-olds from 1990 to 2023. Both sexes follow a broadly parallel, inverted-U trajectory: rates climbed to a pronounced peak around 2002, declined steadily through 2020, and have since tilted upward again. Throughout the entire interval, males maintained a consistently higher plateau than females, underscoring a marked gender gap that persists even as the overall curve shifts.

### 3.2 Joinpoint regression

**Figure 4:**
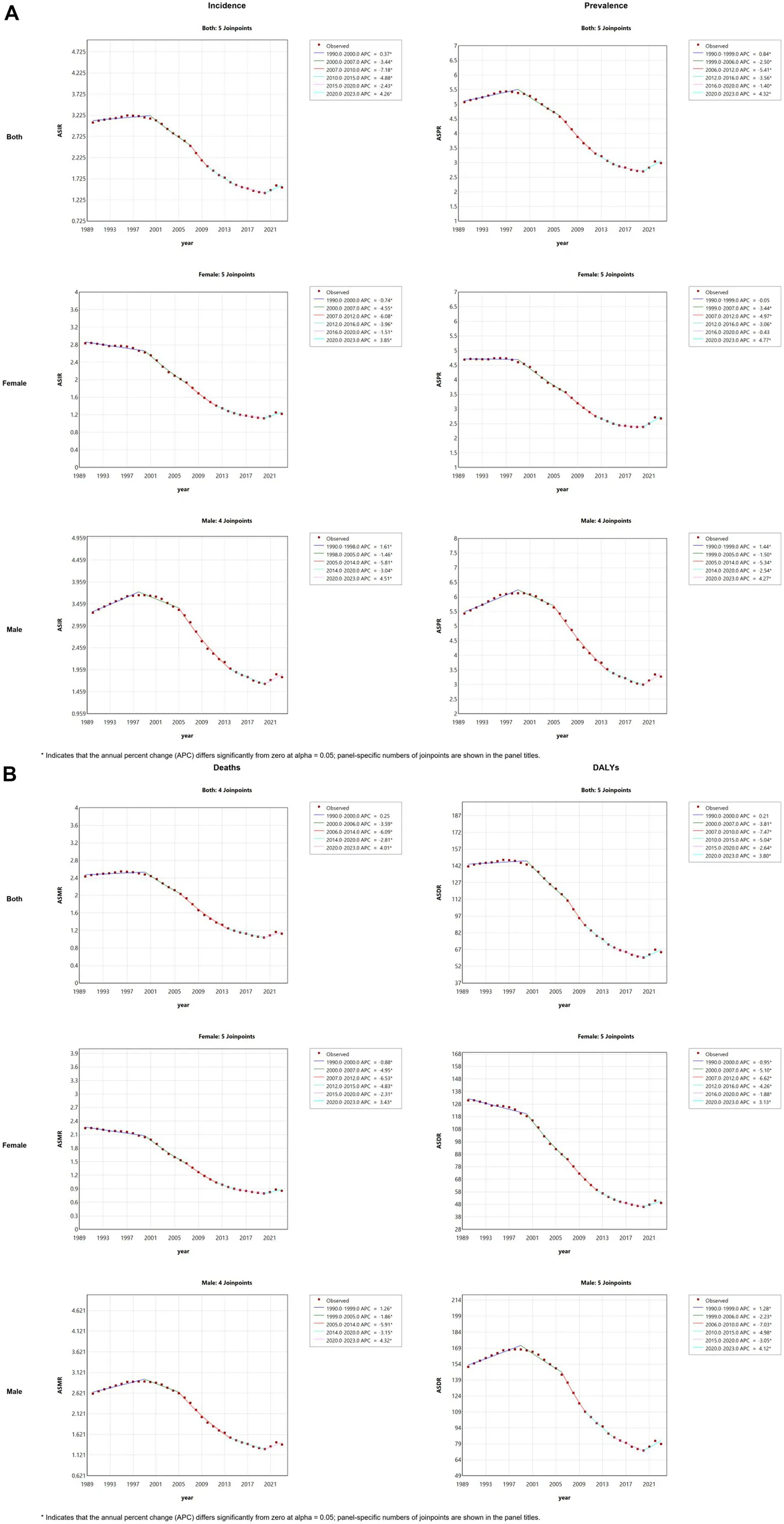
Joinpoint regression analysis results: **(A)** Incidence and prevalence; **(B)** Mortality and DALYs.

Joinpoint modeling (Figure 4) decomposed the 1990–2023 trajectories of age-standardized lung cancer metrics among Chinese individuals aged 15–39 years into distinct linear segments. In males, ASIR increased by +1.61% per year (95% CI 1.02–2.20) during 1990–1998, followed by declines of −1.46% (−2.23 to −0.68) during 1998–2005, −5.81% (−6.23 to −5.39) during 2005–2014, and −3.04% (−3.81 to −2.27) during 2014–2020. This trend reversed after 2020, with ASIR increasing by +4.51% per year (2.12–6.94) during 2020–2023. In females, ASIR declined by −0.74% (−1.03 to −0.44) during 1990–2000, −4.55% (−5.04 to −4.05) during 2000–2007, −6.08% (−6.91 to −5.24) during 2007–2012, −3.96% (−5.15 to −2.76) during 2012–2016, and −1.51% (−2.75 to −0.25) during 2016–2020, before increasing by +3.85% (2.06–5.67) during 2020–2023. ASPR, ASMR, and ASDR showed broadly similar temporal patterns (Table 1), characterized by an overall decline from 1990 to 2023 but a significant reversal toward increasing rates after 2020 in both sexes. Over the entire study period, the declines were more pronounced in females than in males across all four metrics, although the magnitude of the recent increase varied by sex and metric.

**Table 1:** Joinpoint regression analysis of age-standardized lung cancer burden metrics among Chinese adolescents and young adults aged 15–39 years, 1990–2023.

Sex	Measure	Years	APC (95% CI), %	APC *p*-value	AAPC (95% CI), %	AAPC *p*-value
Both	Incidence	1990–2000	0.37 (0.06–0.69)	0.022	−2.04 (−2.40 to −1.68)	<0.001
Both	Incidence	2000–2007	−3.44 (−3.98 - -2.90)	<0.001	−2.04 (−2.40 to −1.68)	<0.001
Both	Incidence	2007–2010	−7.18 (−9.84 - -4.45)	<0.001	−2.04 (−2.40 to −1.68)	<0.001
Both	Incidence	2010–2015	−4.88 (−5.78 - -3.97)	<0.001	−2.04 (−2.40 to −1.68)	<0.001
Both	Incidence	2015–2020	−2.43 (−3.27 - -1.59)	<0.001	−2.04 (−2.40 to −1.68)	<0.001
Both	Incidence	2020–2023	4.26 (2.48–6.07)	<0.001	−2.04 (−2.40 to −1.68)	<0.001
Male	Incidence	1990–1998	1.61 (1.02–2.20)	<0.001	−1.70 (−2.03 to −1.37)	<0.001
Male	Incidence	1998–2005	−1.46 (−2.23 - -0.68)	<0.001	−1.70 (−2.03 to −1.37)	<0.001
Male	Incidence	2005–2014	−5.81 (−6.23 - -5.39)	<0.001	−1.70 (−2.03 to −1.37)	<0.001
Male	Incidence	2014–2020	−3.04 (−3.81 - -2.27)	<0.001	−1.70 (−2.03 to −1.37)	<0.001
Male	Incidence	2020–2023	4.51 (2.12–6.94)	<0.001	−1.70 (−2.03 to −1.37)	<0.001
Female	Incidence	1990–2000	−0.74 (−1.03 - -0.44)	<0.001	−2.46 (−2.76 to −2.16)	<0.001
Female	Incidence	2000–2007	−4.55 (−5.04 - -4.05)	<0.001	−2.46 (−2.76 to −2.16)	<0.001
Female	Incidence	2007–2012	−6.08 (−6.91 - -5.24)	<0.001	−2.46 (−2.76 to −2.16)	<0.001
Female	Incidence	2012–2016	−3.96 (−5.15 - -2.76)	<0.001	−2.46 (−2.76 to −2.16)	<0.001
Female	Incidence	2016–2020	−1.51 (−2.75 - -0.25)	0.022	−2.46 (−2.76 to −2.16)	<0.001
Female	Incidence	2020–2023	3.85 (2.06–5.67)	<0.001	−2.46 (−2.76 to −2.16)	<0.001
Both	Prevalence	1990–1999	0.84 (0.51–1.17)	<0.001	−1.54 (−1.82 to −1.25)	<0.001
Both	Prevalence	1999–2006	−2.50 (−3.00 - -2.00)	<0.001	−1.54 (−1.82 to −1.25)	<0.001
Both	Prevalence	2006–2012	−5.41 (−5.96 - -4.87)	<0.001	−1.54 (−1.82 to −1.25)	<0.001
Both	Prevalence	2012–2016	−3.56 (−4.78 - -2.33)	<0.001	−1.54 (−1.82 to −1.25)	<0.001
Both	Prevalence	2016–2020	−1.40 (−2.59 - -0.19)	0.026	−1.54 (−1.82 to −1.25)	<0.001
Both	Prevalence	2020–2023	4.32 (2.73–5.94)	<0.001	−1.54 (−1.82 to −1.25)	<0.001
Male	Prevalence	1990–1999	1.44 (1.03–1.85)	<0.001	−1.46 (−1.74 to −1.18)	<0.001
Male	Prevalence	1999–2005	−1.50 (−2.36 - -0.63)	0.002	−1.46 (−1.74 to −1.18)	<0.001
Male	Prevalence	2005–2014	−5.34 (−5.68 - -5.00)	<0.001	−1.46 (−1.74 to −1.18)	<0.001
Male	Prevalence	2014–2020	−2.54 (−3.19 - -1.88)	<0.001	−1.46 (−1.74 to −1.18)	<0.001
Male	Prevalence	2020–2023	4.27 (2.36–6.21)	<0.001	−1.46 (−1.74 to −1.18)	<0.001
Female	Prevalence	1990–1999	−0.05 (−0.37–0.27)	0.746	−1.63 (−1.91 to −1.34)	<0.001
Female	Prevalence	1999–2007	−3.44 (−3.79 - -3.08)	<0.001	−1.63 (−1.91 to −1.34)	<0.001
Female	Prevalence	2007–2012	−4.97 (−5.69 - -4.25)	<0.001	−1.63 (−1.91 to −1.34)	<0.001
Female	Prevalence	2012–2016	−3.06 (−4.24 - -1.86)	<0.001	−1.63 (−1.91 to −1.34)	<0.001
Female	Prevalence	2016–2020	−0.43 (−1.69–0.84)	0.482	−1.63 (−1.91 to −1.34)	<0.001
Female	Prevalence	2020–2023	4.77 (3.05–6.52)	<0.001	−1.63 (−1.91 to −1.34)	<0.001
Both	Deaths	1990–2000	0.25 (−0.11–0.61)	0.165	−2.25 (−2.54 to −1.96)	<0.001
Both	Deaths	2000–2006	−3.59 (−4.42 - -2.75)	<0.001	−2.25 (−2.54 to −1.96)	<0.001
Both	Deaths	2006–2014	−6.09 (−6.52 - -5.67)	<0.001	−2.25 (−2.54 to −1.96)	<0.001
Both	Deaths	2014–2020	−2.81 (−3.49 - -2.12)	<0.001	−2.25 (−2.54 to −1.96)	<0.001
Both	Deaths	2020–2023	4.01 (1.93–6.13)	<0.001	−2.25 (−2.54 to −1.96)	<0.001
Male	Deaths	1990–1999	1.26 (0.77–1.74)	<0.001	−1.84 (−2.18 to −1.51)	<0.001
Male	Deaths	1999–2005	−1.86 (−2.87 - -0.84)	0.001	−1.84 (−2.18 to −1.51)	<0.001
Male	Deaths	2005–2014	−5.91 (−6.32 - -5.49)	<0.001	−1.84 (−2.18 to −1.51)	<0.001
Male	Deaths	2014–2020	−3.15 (−3.91 - -2.37)	<0.001	−1.84 (−2.18 to −1.51)	<0.001
Male	Deaths	2020–2023	4.32 (1.92–6.77)	0.001	−1.84 (−2.18 to −1.51)	<0.001
Female	Deaths	1990–2000	−0.88 (−1.16 - -0.58)	<0.001	−2.82 (−3.14 to −2.51)	<0.001
Female	Deaths	2000–2007	−4.95 (−5.44 - -4.46)	<0.001	−2.82 (−3.14 to −2.51)	<0.001
Female	Deaths	2007–2012	−6.53 (−7.34 - -5.72)	<0.001	−2.82 (−3.14 to −2.51)	<0.001
Female	Deaths	2012–2015	−4.83 (−7.12 - -2.49)	<0.001	−2.82 (−3.14 to −2.51)	<0.001
Female	Deaths	2015–2020	−2.31 (−3.08 - -1.54)	<0.001	−2.82 (−3.14 to −2.51)	<0.001
Female	Deaths	2020–2023	3.43 (1.70–5.18)	<0.001	−2.82 (−3.14 to −2.51)	<0.001
Both	DALYs	1990–2000	0.21 (−0.09–0.52)	0.162	−2.29 (−2.64 to −1.93)	<0.001
Both	DALYs	2000–2007	−3.81 (−4.33 - -3.28)	<0.001	−2.29 (−2.64 to −1.93)	<0.001
Both	DALYs	2007–2010	−7.47 (−10.09 - -4.78)	<0.001	−2.29 (−2.64 to −1.93)	<0.001
Both	DALYs	2010–2015	−5.04 (−5.91 - -4.15)	<0.001	−2.29 (−2.64 to −1.93)	<0.001
Both	DALYs	2015–2020	−2.64 (−3.48 - -1.80)	<0.001	−2.29 (−2.64 to −1.93)	<0.001
Both	DALYs	2020–2023	3.80 (2.03–5.60)	<0.001	−2.29 (−2.64 to −1.93)	<0.001
Male	DALYs	1990–1999	1.28 (0.88–1.68)	<0.001	−1.87 (−2.22 to −1.52)	<0.001
Male	DALYs	1999–2006	−2.23 (−2.84 - -1.62)	<0.001	−1.87 (−2.22 to −1.52)	<0.001
Male	DALYs	2006–2010	−7.03 (−8.60 - -5.44)	<0.001	−1.87 (−2.22 to −1.52)	<0.001
Male	DALYs	2010–2015	−4.98 (−5.94 - -4.00)	<0.001	−1.87 (−2.22 to −1.52)	<0.001
Male	DALYs	2015–2020	−3.05 (−3.95 - -2.14)	<0.001	−1.87 (−2.22 to −1.52)	<0.001
Male	DALYs	2020–2023	4.12 (2.12–6.16)	<0.001	−1.87 (−2.22 to −1.52)	<0.001
Female	DALYs	1990–2000	−0.95 (−1.25 - -0.65)	<0.001	−2.87 (−3.17 to −2.57)	<0.001
Female	DALYs	2000–2007	−5.10 (−5.60 - -4.59)	<0.001	−2.87 (−3.17 to −2.57)	<0.001
Female	DALYs	2007–2012	−6.62 (−7.44 - -5.78)	<0.001	−2.87 (−3.17 to −2.57)	<0.001
Female	DALYs	2012–2016	−4.26 (−5.45 - -3.05)	<0.001	−2.87 (−3.17 to −2.57)	<0.001
Female	DALYs	2016–2020	−1.88 (−3.16 - -0.58)	0.007	−2.87 (−3.17 to −2.57)	<0.001
Female	DALYs	2020–2023	3.13 (1.37–4.93)	0.002	−2.87 (−3.17 to −2.57)	<0.001

APC, annual percent change; AAPC, average annual percent change; CI, confidence interval. The Joinpoint analyses were performed using age-standardized rates for Chinese adolescents and young adults aged 15–39 years, not all-age rates. Segment-specific APCs and full-range AAPCs with 95% CIs were calculated by the Joinpoint Regression Program based on these age-standardized rates.

### 3.3 Age–period–cohort (APC) decomposition

To disentangle the patterns underlying the observed reversals, we fitted an APC model to incidence and DALY rates, excluding prevalence because it is jointly determined by disease occurrence and survival (11). Annual data were aggregated into seven calendar periods (1990–1993, 1994–1998, 1999–2003, 2004–2008, 2009–2013, 2014–2018, and 2019–2023) for five 5-year age groups.

**Figure 5:**
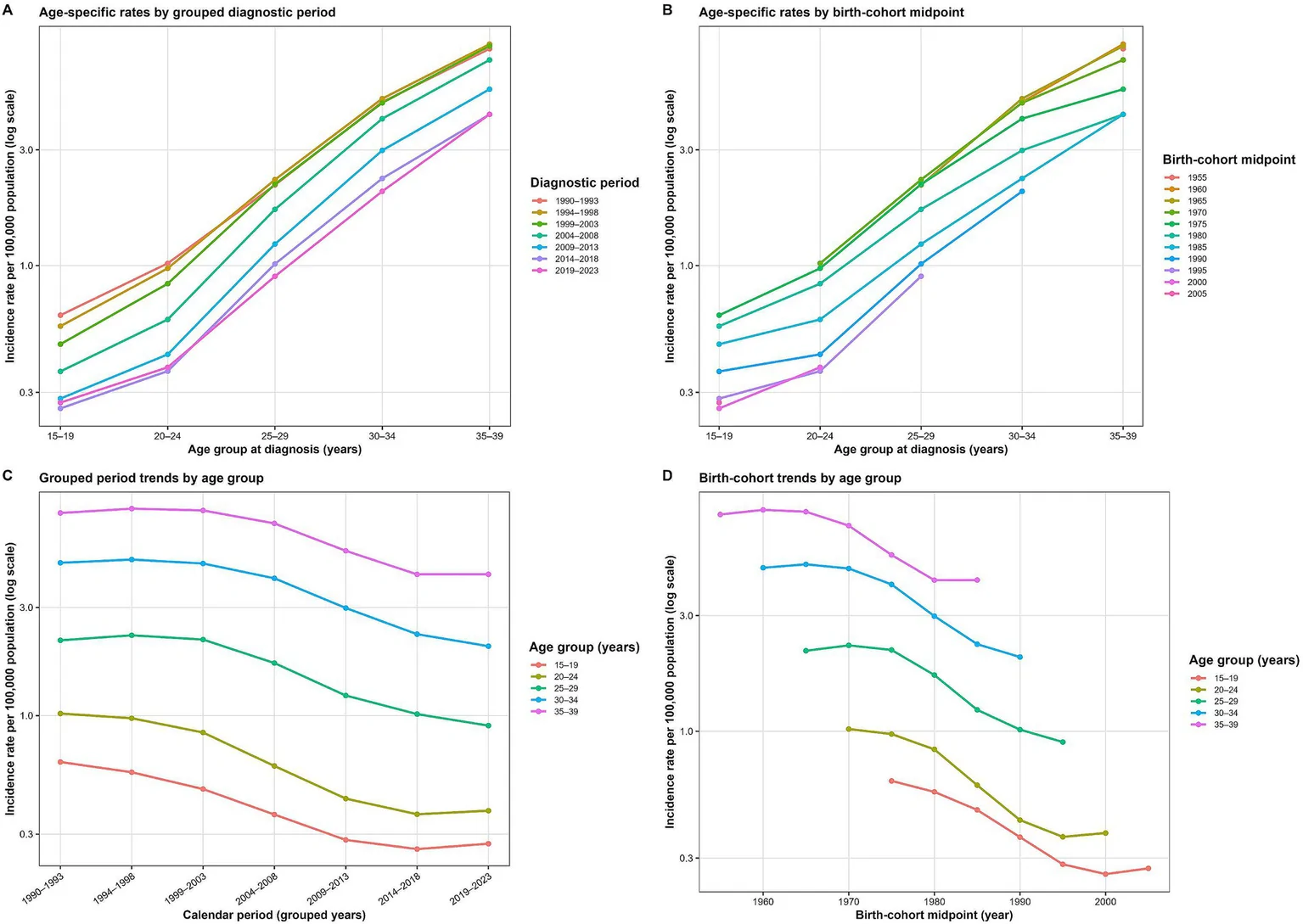
**(A)** Age-specific incidence rates by diagnostic period; **(B)** Age-specific incidence rates by birth cohort; **(C)** Period trends by age group; **(D)** Birth cohort trends by age group.

**Figure 6:**
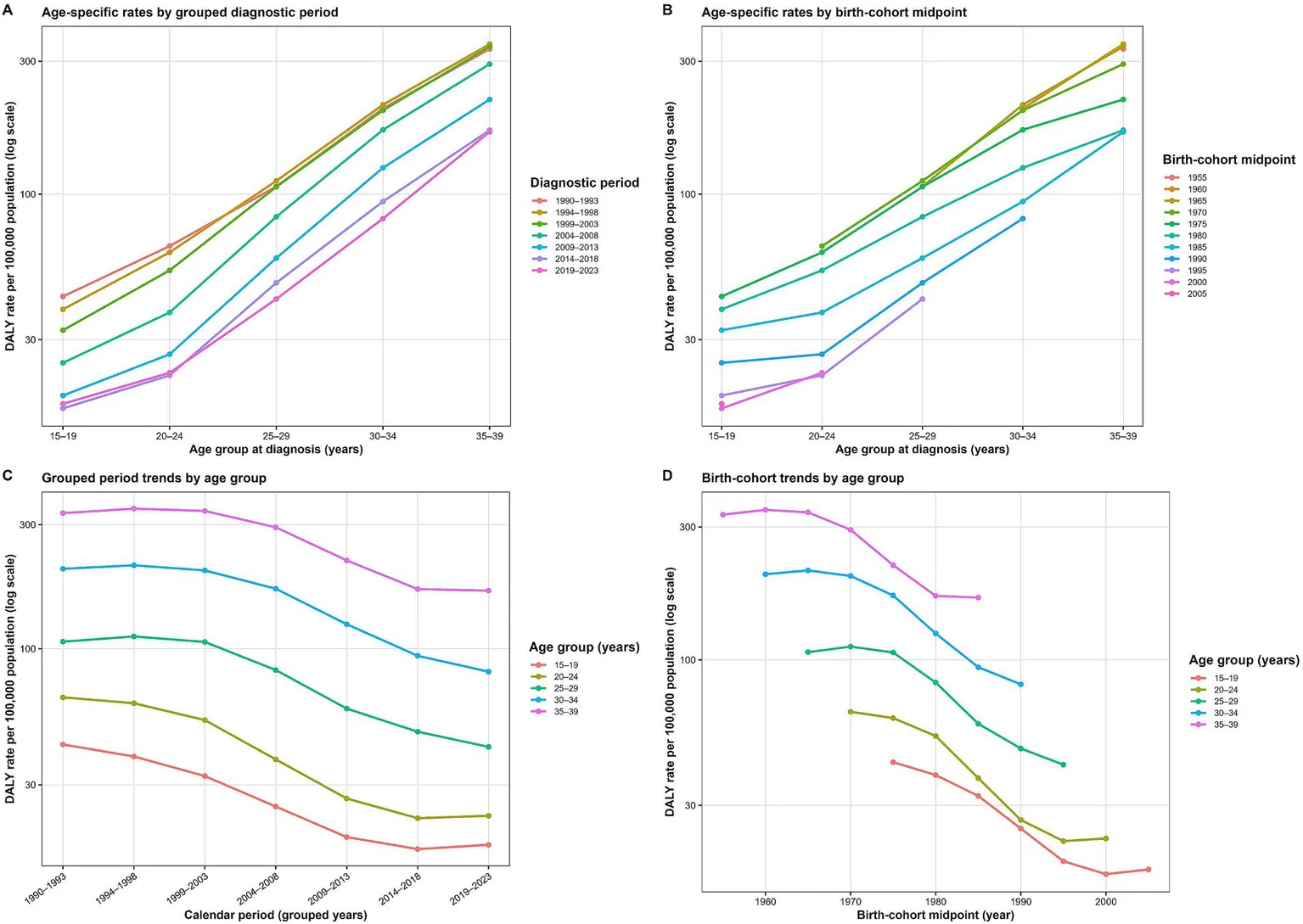
**(A)** Age-specific DALY rates by diagnostic period; **(B)** age-specific DALY rates by birth cohort; **(C)** period trends by age group; **(D)** birth cohort trends by age group.

Figures 5A, 6A show that incidence and DALY rates increased markedly from ages 15–19 to 35–39 years. Figures 5B, 6B indicate progressively lower rates among more recent birth cohorts. Figures 5C,D, 6C,D show generally declining period and cohort trends, although some age groups exhibited flattening or a slight rebound during 2019–2023.

**Figure 7:**
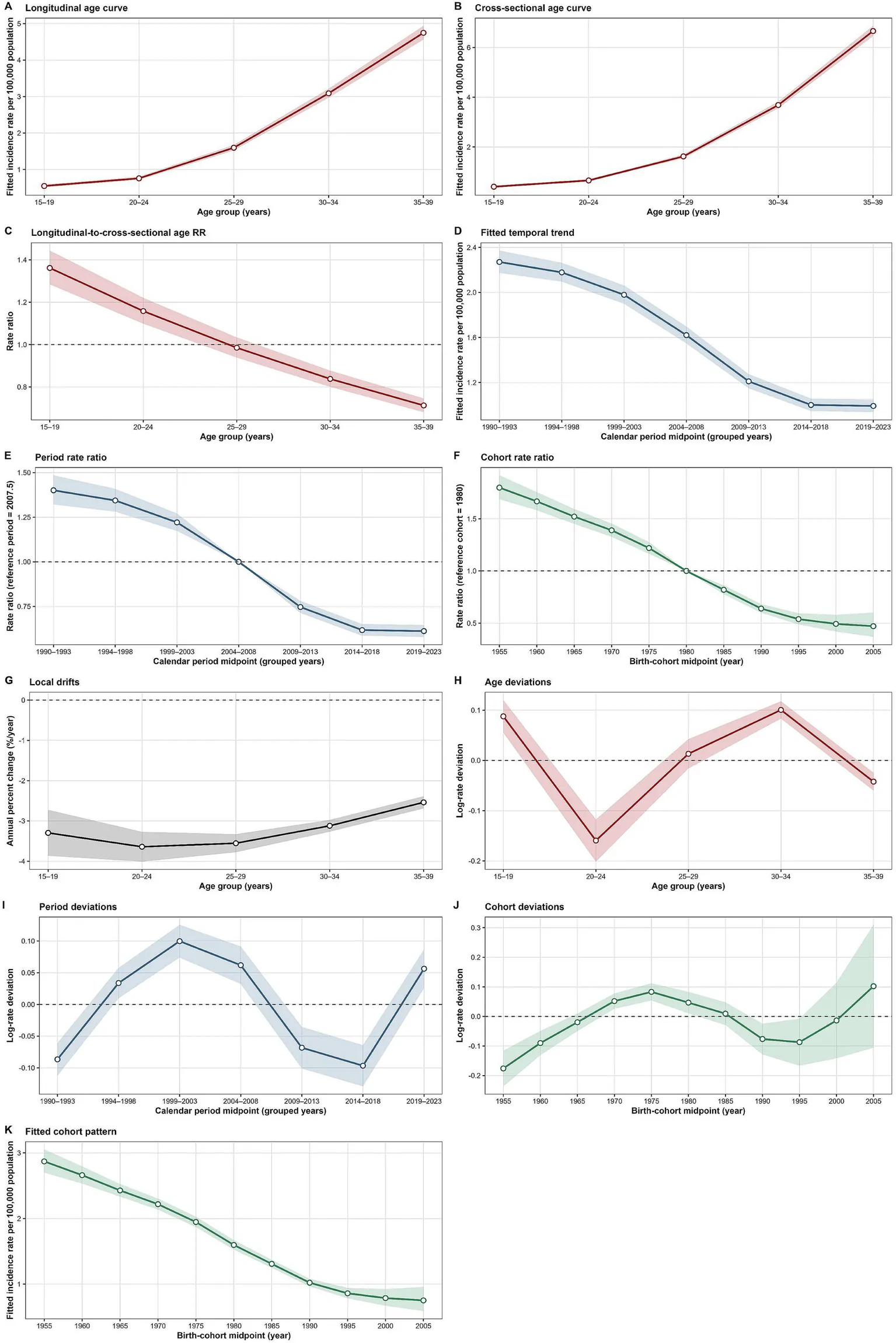
Parameter estimation results of the age–period–cohort (APC) model (based on incidence rates). **(A–F)** represent the longitudinal age curve, cross-sectional age curve, longitudinal-to-cross-sectional age rate ratio, fitted temporal trend, period rate ratio, and cohort rate ratio, respectively. **(G–K)** represent local drifts, age deviations, period deviations, cohort deviations, and fitted cohort pattern, respectively. Rates are expressed per 100,000 population. Shaded areas indicate 95% CIs; dashed lines indicate null values. The reference period was 2007.5 and the reference cohort was 1980.

Figure 7 summarizes the APC decomposition of lung cancer incidence:

1. Longitudinal age curve: Incidence increased steadily with age, particularly after age 30 years.
2. Cross-sectional age curve: Incidence showed a similar age-related increase.
3. Long-vs-cross RR: The ratio declined from 1.36 at ages 15–19 to 0.71 at ages 35–39.
4. Fitted temporal trend: The fitted incidence rate declined from 2.27 to 0.99 per 100,000 between 1990–1993 and 2019–2023.
5. Period RR: Relative to 2004–2008, the RR declined from approximately 1.40 in 1990–1993 to 0.61 in 2019–2023.
6. Cohort RR: Relative to the 1980 cohort, the RR declined progressively to 0.47 for the 2005 cohort.
7. Local drifts: Annual changes were negative across all age groups, ranging from −3.64 to −2.54%.
8. Deviations: Age, period, and cohort deviations indicated modest non-linear fluctuations around the overall downward trends; estimates for the latest cohorts were imprecise.
9. Fitted cohort pattern: Successive birth cohorts generally showed progressively lower fitted incidence rates.

**Figure 8:**
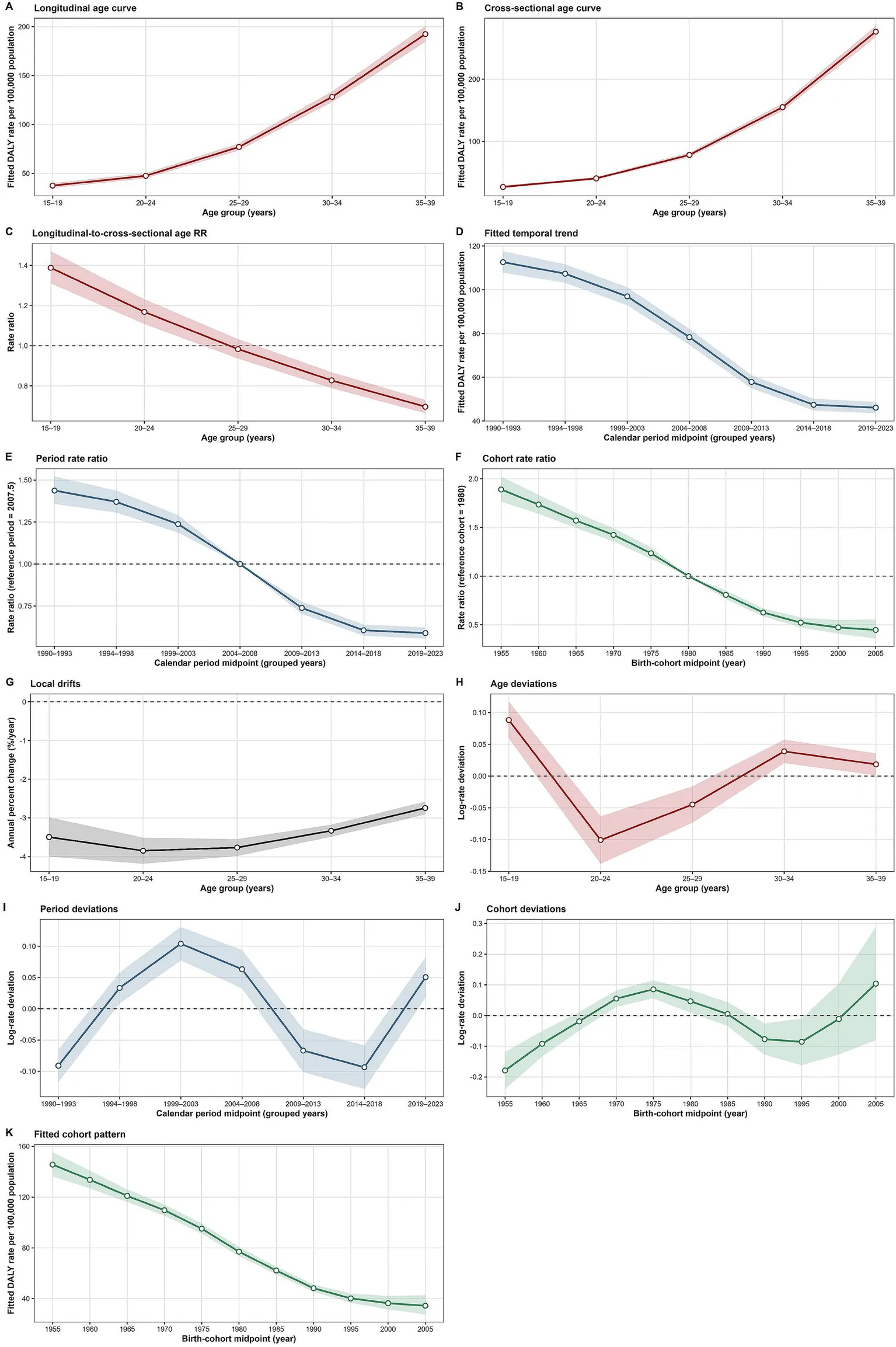
Parameter estimation results of the age–period–cohort (APC) model (based on DALY rates). **(A–F)** Represent the longitudinal age curve, cross-sectional age curve, longitudinal-to-cross-sectional age rate ratio, fitted temporal trend, period rate ratio, and cohort rate ratio, respectively. **(G–K)** Represent local drifts, age deviations, period deviations, cohort deviations, and fitted cohort pattern, respectively. DALY rates are expressed per 100,000 population. Shaded areas indicate 95% CIs; dashed lines indicate null values. The reference period was 2007.5 and the reference cohort was 1980.

The APC decomposition of DALY rates yielded broadly similar patterns (Figure 8), including increasing rates with age and declining period and cohort RRs. These findings indicate consistent population-level age, period, and cohort patterns for incidence and DALYs but do not establish their underlying causal mechanisms.

### 3.4 BAPC projection

**Figure 9:**
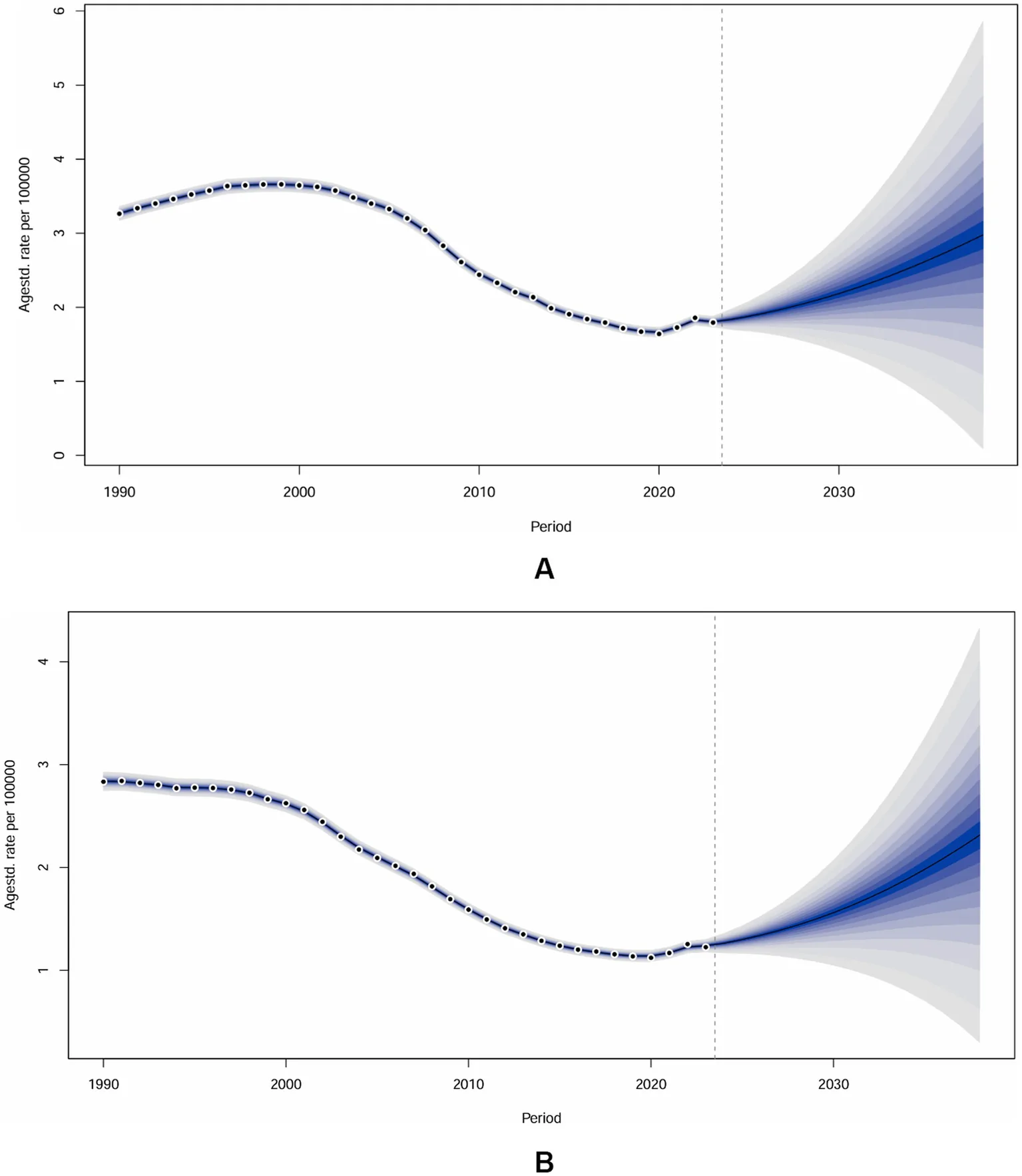
Age-standardized incidence rates **(A)** Males; **(B)** Females.

**Figure 10:**
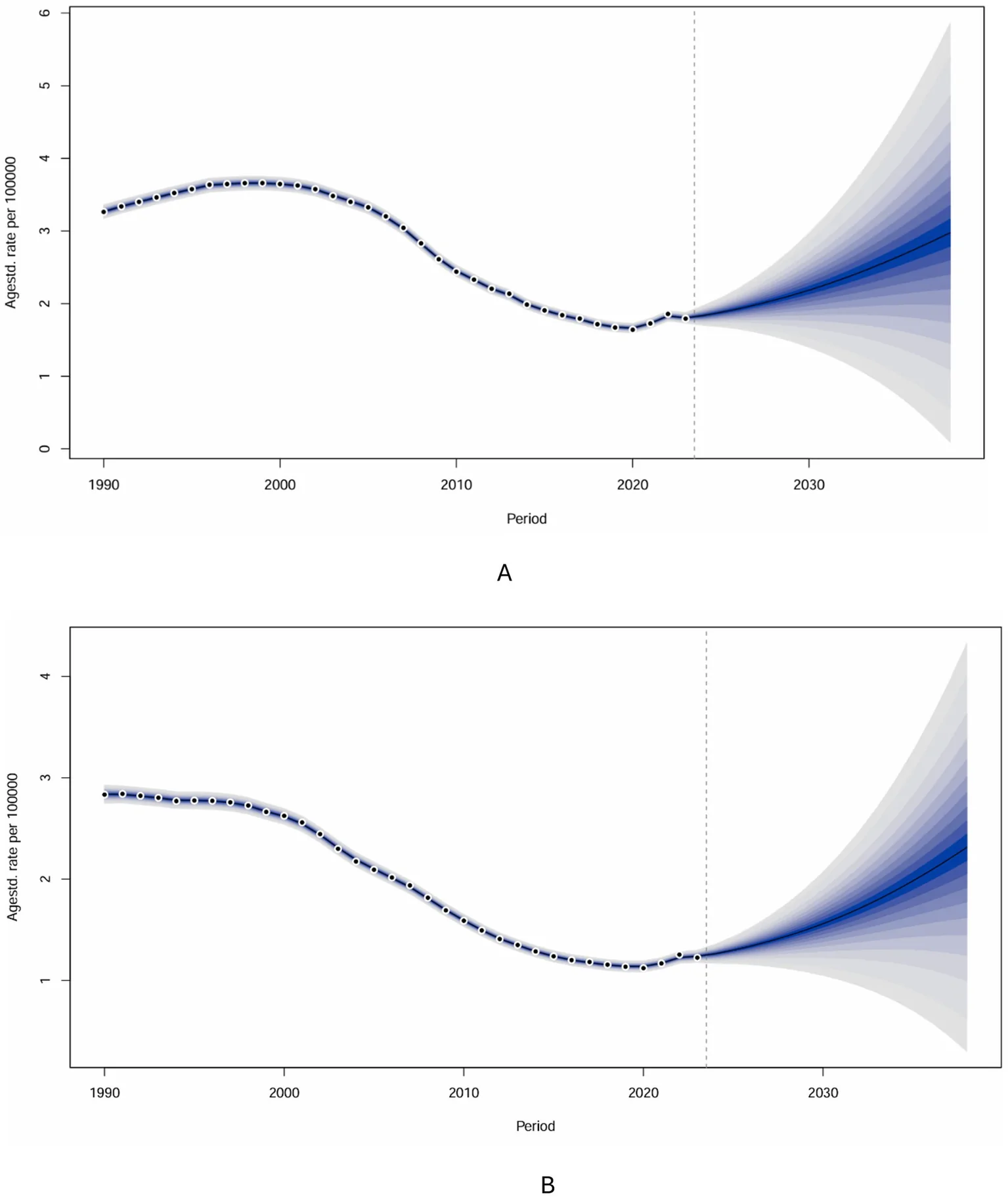
Age-standardized prevalence rates: **(A)** Males; **(B)** Females.

**Figure 11:**
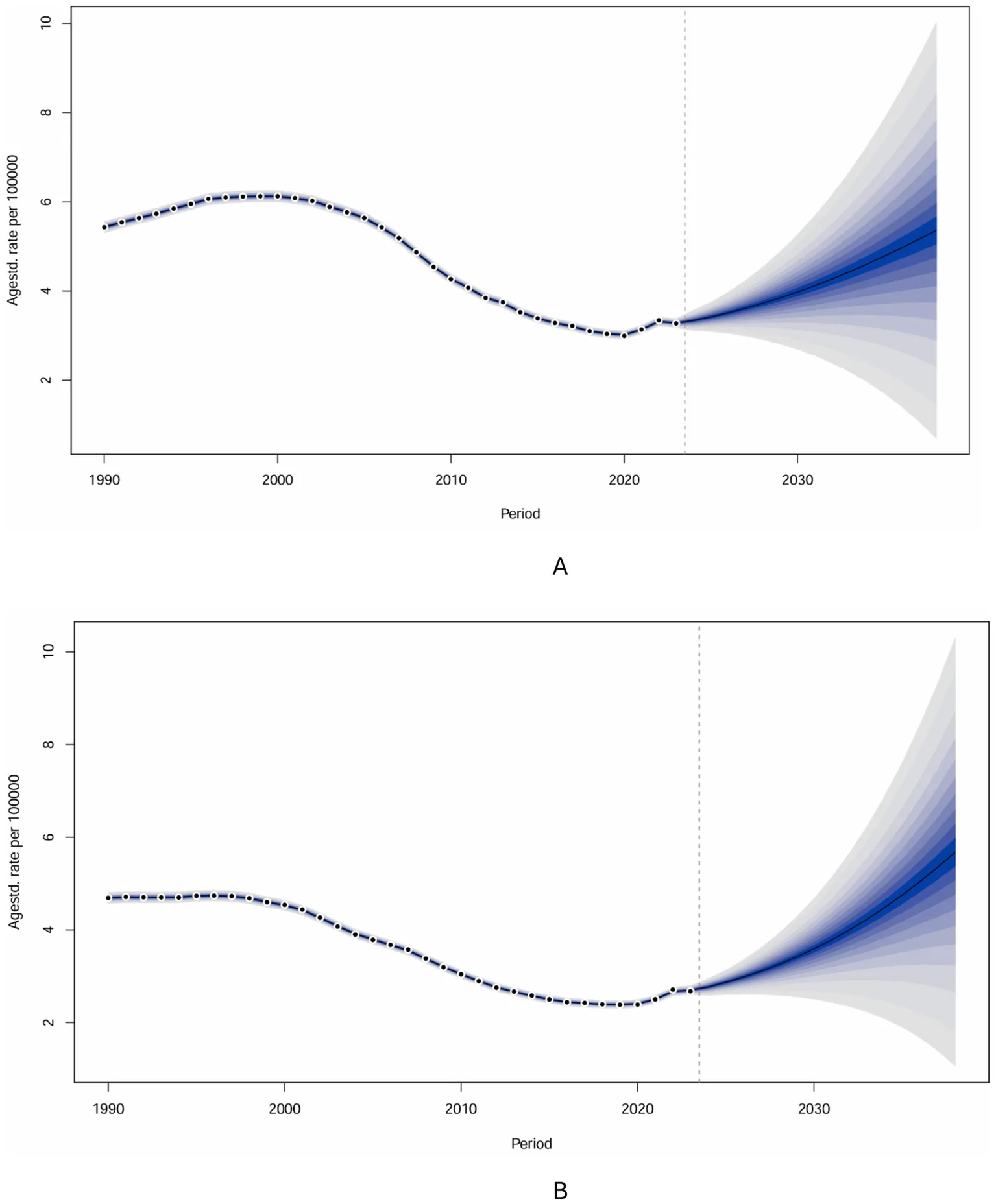
Age-standardized mortality rates **(A)** males; **(B)** females.

**Figure 12:**
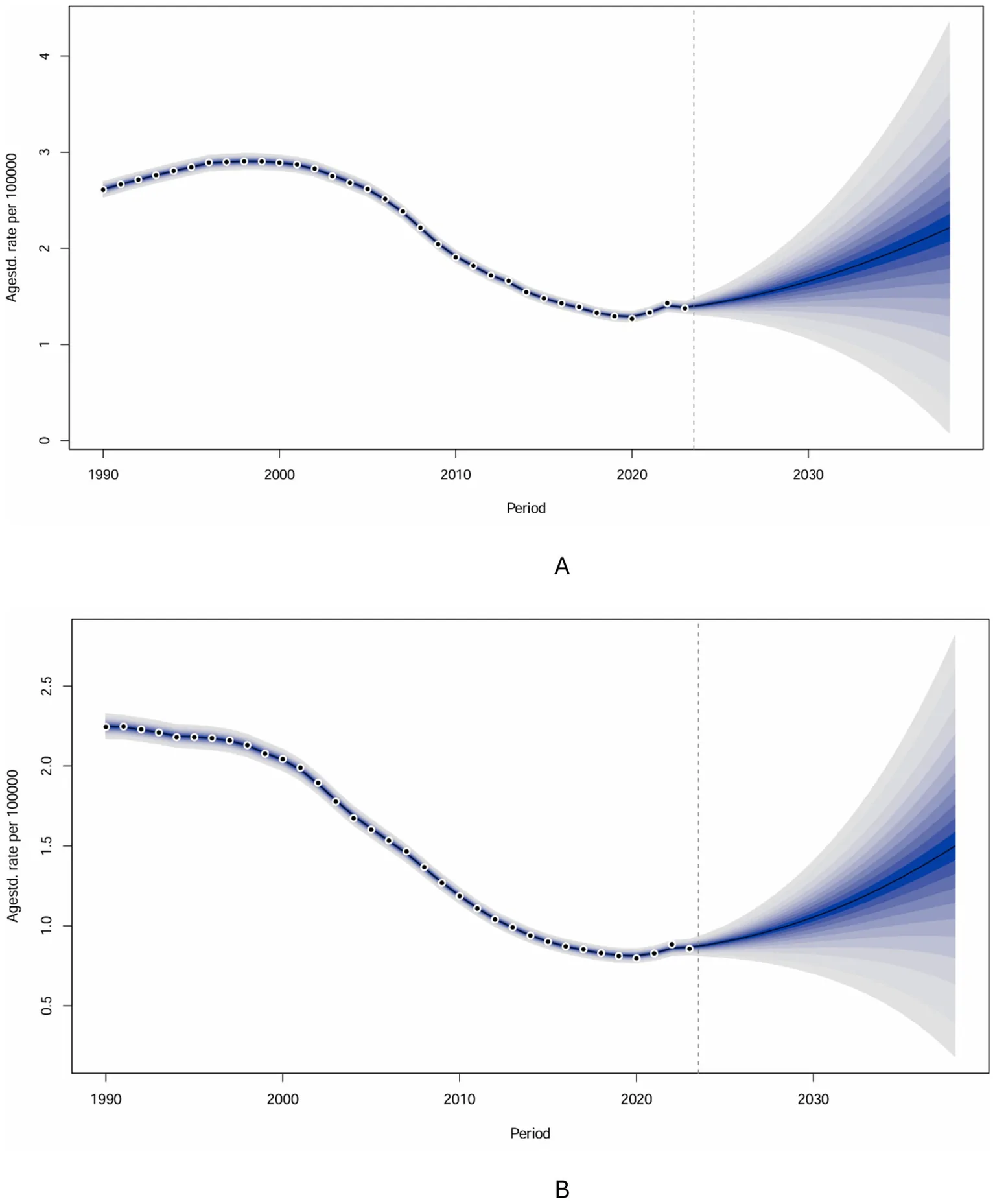
Age-standardized DALY rates **(A)** males; **(B)** females.

Using the Bayesian age-period-cohort (BAPC) model to predict the age-standardized incidence, prevalence, mortality, and DALY rates of lung cancer among young adults aged 15–39 years over the next 15 years(The outputs are presented in Table 2), the results indicate that between 2023 and 2038, the incidence of lung cancer among males in the 15–39 age group will increase from 1.81/100,000 (95% UI: 1.76–1.85) in 2023 to 2.98/100,000 (95% UI: 0.10–5.86) in 2038 (Figure 9A), while female incidence will rise from 1.24/100,000 (95% UI: 1.20–1.28) in 2023 to 2.31/100,000 (95% UI: 0.30–4.32) in 2038 (Figure 9B). Male prevalence will increase from 3.28/100,000 (95% UI: 3.22–3.34) in 2023 to 5.36/100,000 (95% UI: 0.71–10.01) in 2038 (Figure 10A), and female prevalence will rise from 2.70/100,000 (95% UI: 2.64–2.76) to 5.68/100,000 (95% UI: 1.07–10.29) (Figure 10B). Male mortality will increase from 1.39/100,000 (95% UI: 1.35–1.43) in 2023 to 2.21/100,000 (95% UI: 0.08–4.35) in 2038 (Figure 11A), whereas female mortality will rise from 0.87/100,000 (95% UI: 0.84–0.90) in 2023 to 1.50/100,000 (95% UI: 0.18–2.81) in 2038 (Figure 11B). The male DALY rate will increase from 78.9/100,000 (95% UI: 78.6–79.2) in 2023 to 101.3/100,000 (95% UI: 18.7–183.9) in 2038 (Figure 12A), and the female DALY rate will rise from 49.2/100,000 (95% UI: 49.0–49.5) to 56.5/100,000 (95% UI: 11.5–101.6) (Figure 12B).

**Table 2:** BAPC-fitted and projected age-standardized rates of lung cancer among Chinese individuals aged 15–39 years, 2023 and 2038.

Measure	Sex	2023 fitted rate (95% UI)	2038 projected rate (95% UI)
ASIR	Male	1.81 (1.76–1.85)	2.98 (0.10–5.86)
ASIR	Female	1.24 (1.20–1.28)	2.31 (0.30–4.32)
ASIR	Both sexes	1.53 (1.50–1.56)	2.54 (0.35–4.74)
ASPR	Male	3.28 (3.22–3.34)	5.36 (0.71–10.01)
ASPR	Female	2.70 (2.64–2.76)	5.68 (1.07–10.29)
ASPR	Both sexes	3.00 (2.95–3.04)	5.19 (1.12–9.25)
ASMR	Male	1.39 (1.35–1.43)	2.21 (0.08–4.35)
ASMR	Female	0.87 (0.84–0.90)	1.50 (0.18–2.81)
ASMR	Both sexes	1.14 (1.11–1.16)	1.83 (0.24–3.43)
ASDR	Male	78.91 (78.58–79.24)	101.34 (18.77–183.91)
ASDR	Female	49.24 (48.97–49.51)	56.51 (11.47–101.56)
ASDR	Both sexes	64.71 (64.49–64.93)	79.77 (18.95–140.60)

Values are rates per 100,000 population. ASIR, age-standardized incidence rate; ASPR, age-standardized prevalence rate; ASMR, age-standardized mortality rate; ASDR, age-standardized disability-adjusted life-year rate; BAPC, Bayesian age-period-cohort; UI, uncertainty interval. The 2023 estimates are posterior fitted values, whereas the 2038 estimates are BAPC projections. The intervals are approximate 95% Bayesian credible intervals derived from the posterior marginal distributions under the INLA Gaussian approximation; they are model-based and are not the original GBD uncertainty intervals. Rates were age-standardized using the GBD 2023 standard-population weights for ages 15–39 years, renormalized within this age range. Values do not represent absolute case counts.

Values are projected age-standardized rates per 100,000 population in 2038. The main model used default RW2 priors for age, period, and cohort effects with an iid overdispersion term. Sensitivity models used scaled RW2 priors, removed the overdispersion term, or removed the cohort effect. Directional consistency indicates whether the 2023–2038 projection direction was consistent with the main model. For ASDR, the no-overdispersion model produced divergent projections and unstable fit statistics; therefore, DALY projections were interpreted cautiously (Table 3).

**Table 3:** Sensitivity analysis of BAPC projections under alternative model specifications.

Outcome	Sex	Main model 2038	Scaled RW2 2038	No overdisperssion 2038	No cohort effect 2038	Directional consistency
ASIR	Both	2.543	2.531	2.581	1.825	Consistent
ASIR	Male	2.977	2.934	2.972	2.273	Consistent
ASIR	Female	2.312	2.287	2.494	1.458	Consistent
ASPR	Both	5.185	5.165	4.947	4.001	Consistent
ASPR	Male	5.359	5.282	5.199	4.377	Consistent
ASPR	Female	5.680	5.641	6.135	3.816	Consistent
ASMR	Both	1.832	1.823	1.915	1.302	Consistent
ASMR	Male	2.213	2.179	2.266	1.695	Consistent
ASMR	Female	1.499	1.477	1.596	0.954	Consistent
ASDR	Both	79.775	80.357	44.456	73.127	Partly consistent
ASDR	Male	101.338	101.567	51.017	91.592	Partly consistent
ASDR	Female	56.511	57.417	41.354	53.962	Partly consistent

### 3.5 ARIMA projection

**Figure 13:**
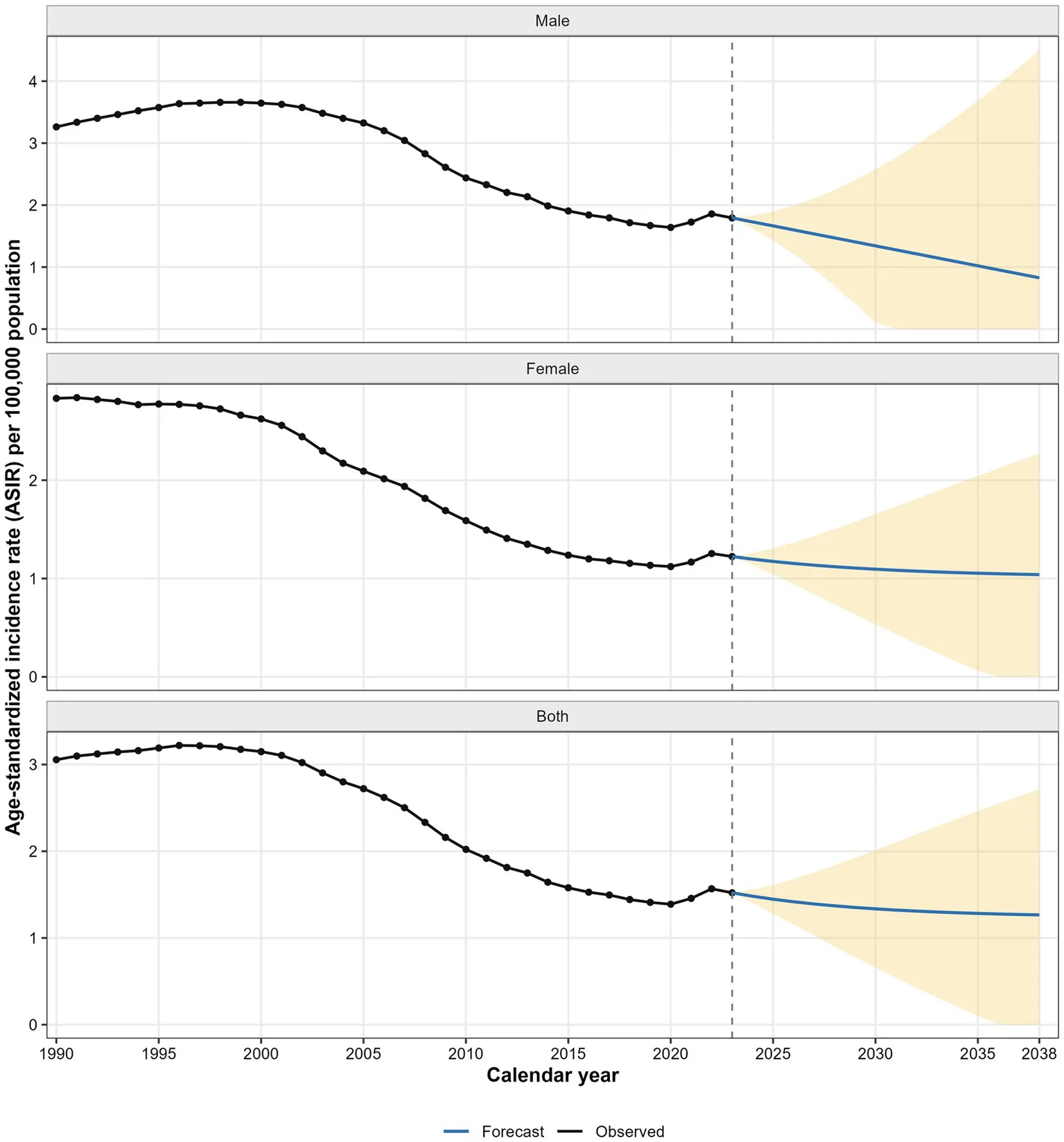
ARIMA model-predicted age-standardized incidence rates: (males, females, both).

**Figure 14:**
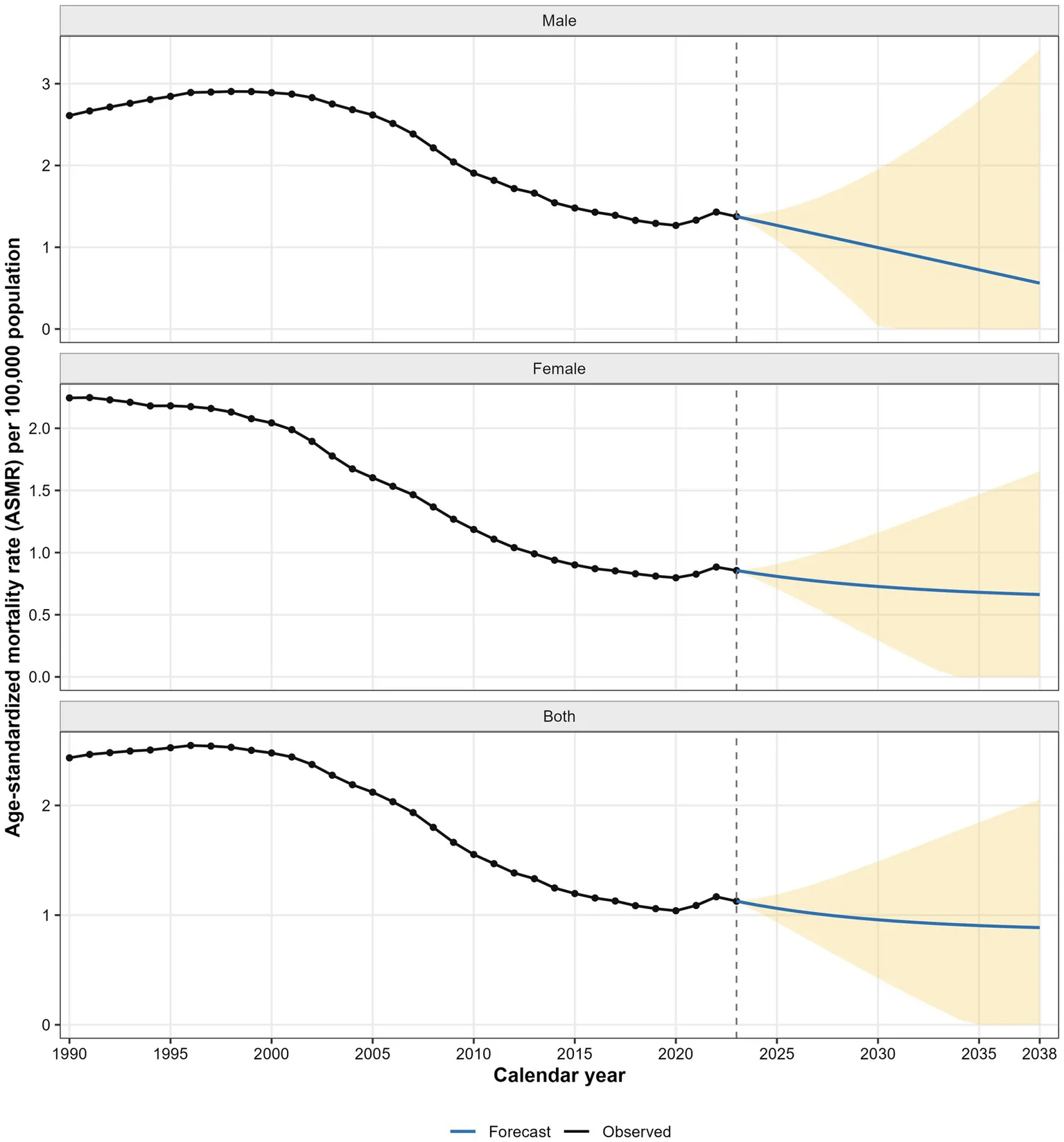
ARIMA model-predicted age-standardized mortality rates: (males–both).

Fitting non-seasonal ARIMA models selected using the auto.arima procedure to the 1990–2023 Chinese lung cancer series for 15- to 39-year-olds yielded sex-specific model orders: ARIMA (0,2,0) for males and ARIMA (1,1,0) for females and both sexes for both incidence and mortality. Both ASIR and ASMR were projected to show modest declines over the next 15 years, with ASIR falling from 1.52 to 1.27 per 100,000 and ASMR from 1.13 to 0.89 by 2038 in both sexes combined, although the prediction intervals were wide and the post-2023 trajectories were relatively flat (Figures 13, 14). The 2038 95% prediction intervals were −0.19 to 2.72 for ASIR and −0.28 to 2.05 for ASMR; lower bounds below zero were truncated at zero in the figures. For ASIR, the estimated AR (1) coefficients were 0.8747 (SE = 0.0715) for females and 0.8601 (SE = 0.0781) for both sexes combined. For ASMR, the corresponding AR (1) coefficients were 0.8948 (SE = 0.0642) and 0.8735 (SE = 0.0735), respectively. No AR or MA coefficients were estimated for the male ARIMA (0,2,0) models.

In rolling-origin validation over 2018–2023, with the model order reselected at each expanding-window step, the selected ARIMA incidence models yielded MAEs of 0.073, 0.041, and 0.056 per 100,000 for males, females, and both sexes, respectively, with corresponding RMSEs of 0.110, 0.059, and 0.074. The selected mortality models yielded MAEs of 0.057, 0.029, and 0.042 and RMSEs of 0.086, 0.042, and 0.056, respectively. The empirical coverage rate of the 95% prediction intervals was 66.7% for all sex–measure series.

## 4 Discussion

Over the past three decades, the lung cancer burden among Chinese adolescents and young adults has followed a broadly downward track, yet our analysis shows that this decline stalled around 2018 and has since reversed. The uptick is neither artefact nor single-cause phenomenon.

Joinpoint regression revealed that, although the age-standardized incidence, prevalence, mortality, and DALY rates of lung cancer in 15-39-year-old Chinese generally declined between 1990 and the late 2010s, each metric showed a recent upward turn around 2020, with varying annual increases thereafter. The male excess in absolute risk persisted, but the post-2020 upswing was evident in both sexes. Part of the increase in ASPR may reflect changes in diagnostic intensity, treatment availability, and survival rather than a true increase in disease onset alone. The inflection also coincided with the COVID-19 pandemic. Previous studies have suggested potential short- and long-term interactions between COVID-19 and lung cancer outcomes (15), but these findings were derived from populations and clinical contexts different from the present GBD-based ecological analysis. Therefore, our data cannot determine whether SARS-CoV-2 infection or pandemic-related healthcare disruptions contributed to the observed trends. Disentangling diagnostic drift, therapeutic advancement, healthcare disruption, and potential viral effects will require prospective cohorts with individual-level imaging, treatment, infection-history, histological, and stage information, as well as longer post-pandemic follow-up.

Within the APC framework, the longitudinal age curve shows a near-logarithmic rise from the youngest to the oldest AYA stratum, with the steepest acceleration between 30 and 39 years, suggesting that—even within this narrow 15-year window—age remains a strong proximate correlate of lung cancer risk. Cross-sectional slices replicate the gradient, but small yet systematic divergences (long-vs-cross RR ≠ 1) suggest additional period and cohort influences. Encouragingly, successive birth generations born after 1985 carry progressively lower cohort-specific risk, a pattern consistent with potential benefits from tobacco-control policies enacted in the 2000s and cleaner household energy transitions, although causal attribution cannot be established from these ecological data. Nevertheless, because China’s population is ageing at an unprecedented pace—projections indicate 400 million residents will be ≥65 years old by 2050, including 150 million ≥80 years—the absolute number of early-onset cases may continue to climb even if age-standardized rates fall. The AYA upturn we document may therefore signal a larger future burden as these cohorts enter later life, underscoring the urgency of primordial prevention starting in adolescence and of capacity planning that anticipates both demographic weight and epidemiological momentum (16).

Projecting the lung cancer burden among Chinese 15- to 39-year-olds to 2038, the two modeling frameworks produced divergent scenarios. The BAPC model, which accounts for age, period, and cohort structures, projected a gentle but persistent increase: by 2038, male and female ASIRs were projected to reach 2.98 and 2.31 per 100,000, respectively, with parallel increases in prevalence, mortality, and DALY rates. In contrast, the ARIMA model suggested modest declines or relatively flat trajectories for ASIR and ASMR, with wide prediction intervals. Expanding-window rolling-origin validation yielded relatively small one-year-ahead errors, but the empirical coverage of the 95% prediction intervals was 66.7%, indicating possible undercoverage of forecast uncertainty. Because this study focused on long-term age–period–cohort dynamics, BAPC was designated as the primary forecasting framework, whereas ARIMA was retained as a complementary short-term scenario (17). The divergence between models was interpreted as model-based forecasting uncertainty.

Tobacco remains an important background risk factor: male smoking prevalence in the 15–39 cohort, though lower than in older generations, is still higher than that of females, which may partly explain the persistently higher incidence, mortality, and DALY rates observed in males (18). Ambient fine-particulate pollution may also contribute to the long-term risk profile. Despite the 2013 “Air Pollution Prevention and Control Action Plan,” population-weighted PM₂.₅ in China fell only to 42 μg/m^3^ in 2021, which remained approximately four times the World Health Organization (WHO) guideline, and longitudinal cohort studies have shown that every 10-μg/m^3^ increment in PM₂.₅ is associated with a 16% excess lung cancer risk in genetically susceptible young adults (19, 20). However, because PM₂.₅ levels in China have declined substantially since 2013, air pollution should not be interpreted as evidence of recent exposure worsening; rather, given the long latency of lung cancer, earlier-life and cumulative exposure may still influence current disease patterns. Finally, changes in diagnostic intensity may have amplified the observed signal; however, organized low-dose computed tomography (CT) screening in China primarily targets older high-risk adults rather than the 15-39-year AYA population. Therefore, any contribution of CT use to the recent increase in AYA lung cancer is likely to reflect incidental or opportunistic detection and remains speculative (21). Taken together, these factors may help explain the persistently higher burden in males and the 35–39 subgroup, but they should be interpreted as plausible hypotheses rather than confirmed causal drivers of the post-2020 rebound. Because this study used population-level GBD estimates, it cannot establish individual-level associations between risk factors and lung cancer outcomes; therefore, the interpretations regarding tobacco, pollution, and screening should be considered speculative.

With tobacco prevalence remaining a concern, PM₂.₅ levels still above WHO guideline values despite recent declines, and uncertainty regarding diagnostic intensity in younger adults, a low-slope increase remains possible but should be interpreted cautiously for China’s young adults—slow enough to permit containment if intensified clean-air enforcement and youth-focused cessation programs are deployed now.

Our findings extend the GBD 2021 analysis by Han et al., which examined lung cancer burden among individuals aged 15–45 years worldwide from 1990 to 2021 and reported increasing case numbers but generally declining age-standardized prevalence, mortality, and DALY rates, with a higher burden in men and less favorable patterns in East Asia and some lower-SDI regions (3). In contrast, we focused on Chinese individuals aged 15–39 years, used updated GBD 2023 estimates through 2023, and integrated Joinpoint, APC, BAPC, and ARIMA methods, identifying a post-2020 increase in several age-standardized indicators and projecting trends through 2038. Tian et al.’s multicenter clinical study further showed that young Chinese patients with lung cancer had distinct clinicopathological and molecular features, including lower smoking prevalence and enrichment of actionable alterations such as ERBB2 mutations and ALK rearrangements (22). Whereas their study characterized individual-level tumor biology and treatment-related features, our analysis quantified the population-level burden and temporal trajectory, providing complementary epidemiological evidence for lung cancer among Chinese AYA individuals.

### 4.1 Limitations

This study has several limitations. First, GBD estimates are modeled outputs rather than raw observed counts, and their accuracy and completeness depend on the quality of the underlying vital registration, cancer registry, and survey systems; under-reporting, inconsistent case definitions, and sparse data may introduce uncertainty, particularly for rare outcomes in narrow age groups. Although GBD uncertainty intervals were reported, this uncertainty was not fully propagated through all subsequent trend and forecasting analyses. Second, this ecological analysis cannot establish individual-level associations between lung cancer outcomes and tobacco exposure, air pollution, or screening; these explanations should therefore be considered plausible hypotheses rather than confirmed causal drivers. Third, GBD does not provide histological, stage-specific, smoking-status, or molecular data for individuals aged 15–39 years, precluding analysis of changing proportions of adenocarcinoma and other subtypes, particularly among young never-smokers. Fourth, the data used in this study did not permit provincial, urban–rural, or socioeconomic stratification, preventing assessment of important geographic and social disparities in pollution, smoking prevalence, healthcare access, and diagnostic capacity within China. Fifth, the China-specific design and absence of formal comparisons with Japan, South Korea, or global patterns limit the international generalizability of our findings. Finally, the divergence between BAPC and ARIMA projections highlights the inherent uncertainty of long-term forecasting; neither model can fully capture unforeseen policy interventions, changes in exposure, or technological advances that may alter future trajectories. Absolute counts may be affected by population growth and changes in age structure; therefore, this study primarily interprets changes in age-standardized rates.

## 5 Conclusion

From 1990 to 2023, the lung cancer burden among Chinese adolescents and young adults fell substantially, testimony to decades of tobacco control and gradual air-quality improvements. Since 2020, however, every key metric has turned upward, and Bayesian projection indicates that without additional interventions this upswing will persist through 2038. The resurgence is modest in absolute numbers but sufficient to strain an already over-stretched oncology workforce that must simultaneously prepare for the much larger wave of elderly cases driven by population ageing. Sustaining the earlier downward trajectory will require sharper focus on the AYA decade: expanding smoke-free campuses, enforcing stricter PM₂.₅ targets, and deploying low-dose CT judiciously for high-risk young adults. Developing accurate, cost-effective diagnostics and risk-stratified early-detection algorithms tailored to this age group is no longer optional—it is an urgent investment if China is to avert a new generation of preventable lung cancer deaths.

## Data Availability

Publicly available datasets were analyzed in this study. This data can be found here: https://ghdx.healthdata.org/gbd-2021/sources.

## References

[1] National Cancer Institute Risk Factors: Age Bethesda (MD): National Cancer Institute (2025). Available online at: https://www.cancer.gov/about-cancer/causes-prevention/risk/age (Accessed July 16, 2026).

[2] Fidler-Benaoudia MM Torre LA Bray F Ferlay J Jemal A. Lung cancer incidence in young women vs. young men: a systematic analysis in 40 countries. Int J Cancer. (2020) 147:811–9. doi: 10.1002/ijc.32809, 32020598

[3] Han Z Zhu Z Zhang Z Dong J Yang X Feng J. Global burden of lung cancer in adolescents and adults aged 15–45: analysis of the global burden of disease study (1990–2021). Front Med (Lausanne). (2025) 12:1600662. doi: 10.3389/fmed.2025.1600662, 40636363 PMC12239874

[4] GBD. Causes of death collaborators global burden of 292 causes of death in 204 countries and territories and 660 subnational locations, 1990–2023: a systematic analysis for the global burden of disease study 2023. Lancet. (2023a) 406:1811–72. doi: 10.1016/S0140-6736(25)01917-8, 41092928 PMC12535838

[5] GBD 2023 Demographics Collaborators. Global age-sex-specific all-cause mortality and life expectancy estimates for 204 countries and territories and 660 subnational locations, 1950–2023: a demographic analysis for the global burden of disease study 2023. Lancet. (2025) 406:1731–810. doi: 10.1016/S0140-6736(25)01330-3 41092927 PMC12535839

[6] GBD (2023b). Disease and injury and risk factor collaborators burden of 375 diseases and injuries, risk-attributable burden of 88 risk factors, and healthy life expectancy in 204 countries and territories, including 660 subnational locations, 1990–2023: a systematic analysis for the global burden of disease study 2023 Lancet 2023 406 1873–1922 doi: 10.1016/S0140-6736(25)01637-X 41092926 PMC12535840

[7] Riebler A Held L. Projecting the future burden of cancer: Bayesian age-period-cohort analysis with integrated nested Laplace approximations. Biom J. (2017) 59:531–49. doi: 10.1002/bimj.201500263, 28139001

[8] Jürgens V Ess S Cerny T Vounatsou P. A Bayesian generalized age-period-cohort power model for cancer projections. Stat Med. (2014) 33:4627–36. doi: 10.1002/sim.6248, 24996118

[9] Ahmad OB Boschi-Pinto C Lopez AD Murray CJL Lozano R Inoue M. Age standardization of rates: a new WHO standard. Geneva: World Health Organization; (2001). GPE Discussion Paper Series No. 31.

[10] Kim HJ Fay MP Feuer EJ Midthune DN. Permutation tests for joinpoint regression with applications to cancer rates. Stat Med. (2000) 19:335–51. doi: 10.1002/(SICI)1097-0258(20000215)19:3<335::AID-SIM336>3.0.CO;2-Z, 10649300

[11] Rosenberg PS Check DP Anderson WF. A web tool for age-period-cohort analysis of cancer incidence and mortality rates. Cancer Epidemiol Biomarkers Prev. (2014) 23:2296–302. doi: 10.1158/1055-9965.EPI-14-0300, 25146089 PMC4221491

[12] Holford TR. The estimation of age, period, and cohort effects for vital rates. Biometrics. (1983) 39:311–24. doi: 10.2307/2531004, 6626659

[13] Rue H Martino S Chopin N. Approximate Bayesian inference for latent gaussian models by using integrated nested Laplace approximations. J R Stat Soc Series B Stat Methodol. (2009) 71:319–92. doi: 10.1111/j.1467-9868.2008.00700.x

[14] Schaffer AL Dobbins TA Pearson SA. Interrupted time series analysis using autoregressive integrated moving average (ARIMA) models: a guide for evaluating large-scale health interventions. BMC Med Res Methodol. (2021) 21:58. doi: 10.1186/s12874-021-01235-0 33752604 PMC7986567

[15] Peng YL Wang ZY Zhong RW Mei SQ Liu JQ Tang LB . Association of COVID-19 and lung cancer: short-term and long-term interactions. Cancers (Basel). (2024) 16:304. doi: 10.3390/cancers16020304, 38254793 PMC10813989

[16] Fang EF Scheibye-Knudsen M Jahn HJ Li J Ling L Guo H . A research agenda for aging in China in the 21st century. Ageing Res Rev. (2015) 24:197–205. doi: 10.1016/j.arr.2015.08.003, 26304837 PMC5179143

[17] Trächsel B Rousson V Bulliard JL Locatelli I. Comparison of statistical models to predict age-standardized cancer incidence in Switzerland. Biom J. (2023) 65:e2200046. doi: 10.1002/bimj.202200046, 37078835

[18] Walser T Cui X Yanagawa J Lee JM Heinrich E Lee G . Smoking and lung cancer: the role of inflammation. Proc Am Thorac Soc. (2008) 5:811–5. doi: 10.1513/pats.200809-100TH, 19017734 PMC4080902

[19] Liang F Xiao Q Huang K . The 17-year spatiotemporal trend of PM2.5 and its mortality burden in China. Proc Natl Acad Sci USA. (2020) 117:25601–8. doi: 10.1073/pnas.1919641117 32958653 PMC7568266

[20] Huang Y Zhu M Ji M Fan J Xie J Wei X . Air pollution, genetic factors, and the risk of lung cancer: a prospective study in the UK biobank. Am J Respir Crit Care Med. (2021) 204:817–25. doi: 10.1164/rccm.202011-4063OC, 34252012

[21] Vachani A Carroll NM Simoff MJ Neslund-Dudas C Honda S Greenlee RT . Stage migration and lung cancer incidence after initiation of low-dose computed tomography screening. J Thorac Oncol. (2022) 17:1355–64. doi: 10.1016/j.jtho.2022.08.011, 36087860 PMC9703625

[22] Tian Y Ma R Zhao W Wang S Zhou C Wu W . Comprehensive characterization of early-onset lung cancer in Chinese young adults. Nat Commun. (2025) 16:1976. doi: 10.1038/s41467-025-57309-4, 40000630 PMC11861273

